# Food Insecurity and Its Association With Depressive Symptoms: Evidence From Older Adults in India

**DOI:** 10.1093/geroni/igaf122.2017

**Published:** 2025-12-31

**Authors:** Sindhu Vasireddy

**Affiliations:** Mahindra University, Hyderabad, Telangana, India

## Abstract

This study is novel in examining the association between both the prevalence and incidence of food insecurity and depressive symptoms among older adults in India. For this, we use two waves of data from the Study of Global AGEing and Adult Health (SAGE) conducted in 2007 and 2015. Logistic regression analysis is employed to examine the prevalence of depression, while a discrete-time event history model is used to study the incidence of depression and determine the influence of food insecurity on the onset and offset of depression. We find that severe food insecurity is strongly associated with increased odds of depression, and that this association strengthened across the waves. The incidence models find that relative to those who do not experience any transitions, when people transition from being insecure to secure about food over the waves, they are twice (OR: 2.035, CI: 1.53-2.69) as likely to move out of depression. Similarly, those who experience the onset of food insecurity are more likely to transition into depression over the waves. In addition to health counselors and psychological support, this study fortifies the necessity for policies and interventions targeted to lessen hunger and improve the affordability of food to alleviate depressive symptoms in Indian older adults.

